# Loss of dysferlin or myoferlin results in differential defects in excitation–contraction coupling in mouse skeletal muscle

**DOI:** 10.1038/s41598-021-95378-9

**Published:** 2021-08-05

**Authors:** David Y. Barefield, Jordan J. Sell, Ibrahim Tahtah, Samuel D. Kearns, Elizabeth M. McNally, Alexis R. Demonbreun

**Affiliations:** 1 Center for Genetic Medicine, Feinberg School of Medicine, Northwestern University, 303 E Superior Lurie 5-500, Chicago, IL 60611 USA; 2 Department of Cell and Molecular Physiology, Loyola University Chicago, 2160 S. 1st Ave, Maywood, IL 60153 USA; 3 Department of Pharmacology, Feinberg School of Medicine, Northwestern University, Chicago, IL USA; 4 Center for Genetic Medicine, Northwestern University, 303 E Superior Lurie 5-512, Chicago, IL 60611 USA

**Keywords:** Calcium signalling, Cell biology, Cellular imaging, Mechanisms of disease

## Abstract

Muscular dystrophies are disorders characterized by progressive muscle loss and weakness that are both genotypically and phenotypically heterogenous. Progression of muscle disease arises from impaired regeneration, plasma membrane instability, defective membrane repair, and calcium mishandling. The ferlin protein family, including dysferlin and myoferlin, are calcium-binding, membrane-associated proteins that regulate membrane fusion, trafficking, and tubule formation. Mice lacking dysferlin (Dysf), myoferlin (Myof), and both dysferlin and myoferlin (Fer) on an isogenic inbred 129 background were previously demonstrated that loss of both dysferlin and myoferlin resulted in more severe muscle disease than loss of either gene alone. Furthermore, Fer mice had disordered triad organization with visibly malformed transverse tubules and sarcoplasmic reticulum, suggesting distinct roles of dysferlin and myoferlin. To assess the physiological role of disorganized triads, we now assessed excitation contraction (EC) coupling in these models. We identified differential abnormalities in EC coupling and ryanodine receptor disruption in flexor digitorum brevis myofibers isolated from ferlin mutant mice. We found that loss of dysferlin alone preserved sensitivity for EC coupling and was associated with larger ryanodine receptor clusters compared to wildtype myofibers. Loss of myoferlin alone or together with a loss of dysferlin reduced sensitivity for EC coupling, and produced disorganized and smaller ryanodine receptor cluster size compared to wildtype myofibers. These data reveal impaired EC coupling in Myof and Fer myofibers and slightly potentiated EC coupling in Dysf myofibers. Despite high homology, dysferlin and myoferlin have differential roles in regulating sarcotubular formation and maintenance resulting in unique impairments in calcium handling properties.

## Introduction

Muscular dystrophies are disorders characterized by progressive muscle loss and weakness that often result from loss of function mutations in cytoskeletal and membrane-associated proteins^1–3^. Dysregulation of cytoplasmic calcium is a common pathological feature in many muscular dystrophies and is thought to contribute to disease progression^4–10^. Vertebrate skeletal muscle excitation contraction (EC) coupling occurs at the triad, a structure composed of a transverse-tubule (t-tubule) tightly localized between two sarcoplasmic reticulum cisternae (SR)^4,11,12^. EC coupling relies on a mechanical coupling of the t-tubule associated dihydropyridine receptor (DHPR) and the SR associated ryanodine receptor (RyR)^13–15^. Upon activation, DHPR stimulates RyR to release calcium from the SR into the cytoplasm, resulting in contraction. Closing of the RyR and subsequent calcium reuptake by sarcoplasmic reticulum calcium ATPase (SERCA) initiates muscle relaxation. Alterations in EC coupling can occur from multiple mechanisms including mutations in DHPR/RyR, calcium channel dysregulation, malformation of the triad structure, and perturbed calcium storage/release leading to myopathy^4,5,16–20^.

Dysferlin, a member of the ferlin protein family, is a 230-kDa membrane-associated protein composed of multiple calcium-binding C2 domains and a carboxy-terminal transmembrane domain^21–24^. Dysferlin is highly expressed in mature skeletal myofibers and is expressed at low levels in muscle progenitor myoblast cells^21,23,25,26^. Loss-of-function mutations in dysferlin results in human muscular dystrophy, and mice lacking dysferlin (Dysf) display similar pathological features to the human disorder^22,27–32^. Dysferlin regulates many cellular processes including membrane repair, vesicle trafficking and the development and maintenance of the t-tubule system^4,21,33–37^. Dysferlin localizes to the t-tubule and mice lacking dysferlin have disarrayed, dilated t-tubules that are highly susceptible to injury^4,29,30,35,36,38^. Additional evidence supports a role for dysferlin stabilizing the DHPR in the t-tubule and the ryanodine receptor in the SR, which is essential for proper EC coupling^5,35,36^.

Myoferlin is a homologue of dysferlin with a 76% resemblance at the amino acid level^39^. Similar to dysferlin, myoferlin contains seven C2 domains and a carboxy-terminal transmembrane domain^23,24^. Although myoferlin and dysferlin are homologous to each other, myoferlin is highly expressed in myoblasts with expression levels decreasing as muscle matures^23,25,40,41^. Myoferlin is re-expressed upon muscle injury^42,43^. The absence of myoferlin leads to impaired myoblast fusion and delayed muscle regeneration after injury^41,42^. In addition, adult mice lacking myoferlin (Myof) develop disorganized triads with enlargement of the sarcoplasmic reticulum^30^. However, unlike dysferlin, no clinical forms of muscular dystrophy due to mutations in myoferlin have been reported.

Mice lacking both dysferlin and myoferlin, termed Fer, were previously generated and showed more severe muscle disease than loss of myoferlin or dysferlin alone, suggesting non-redundant roles for these proteins^30,40,44^. Electron microscopy studies of Fer muscle revealed ectopic triad formation in addition to enlargement of the SR and elongation of the t-tubule compartments^30^. This correlated with perturbations in DHPR immunofluorescence staining, showing DHPR aggregates and areas devoid of DHPR fluorescence^30^. Furthermore, Fer muscle developed significantly more sarcotubular aggregates compared to Dysf, Myof, and WT mice^30^. We hypothesized that the severe triad disorganization in Fer mice will lead to defects in excitation–contraction coupling.

Herein, we performed single cell shortening and calcium handling measurements on live myofibers isolated from the flexor digitorum brevis (FDB) muscle from WT, Dysf, Myof, and Fer mice, stimulated at twitch, 40 Hz, and 80 Hz. We found that loss of dysferlin alone caused a small reduction in calcium transient amplitude but did not reduce sensitivity of EC coupling or alter cell shortening, whereas loss of myoferlin alone or in combination with dysferlin caused aberrant EC coupling with smaller calcium transients and slower calcium release kinetics. These differences were noted in ryanodine receptor disorganization, with loss of myoferlin alone or in combination with loss of dysferlin showing the greatest changes in RyR cluster size and distribution. These data indicate that loss of myoferlin and dysferlin result in differential morphological patterns within mature muscle fibers.

## Results

### Loss of Ferlin proteins minimally alters unloaded sarcomere shortening

**Figure 1:**
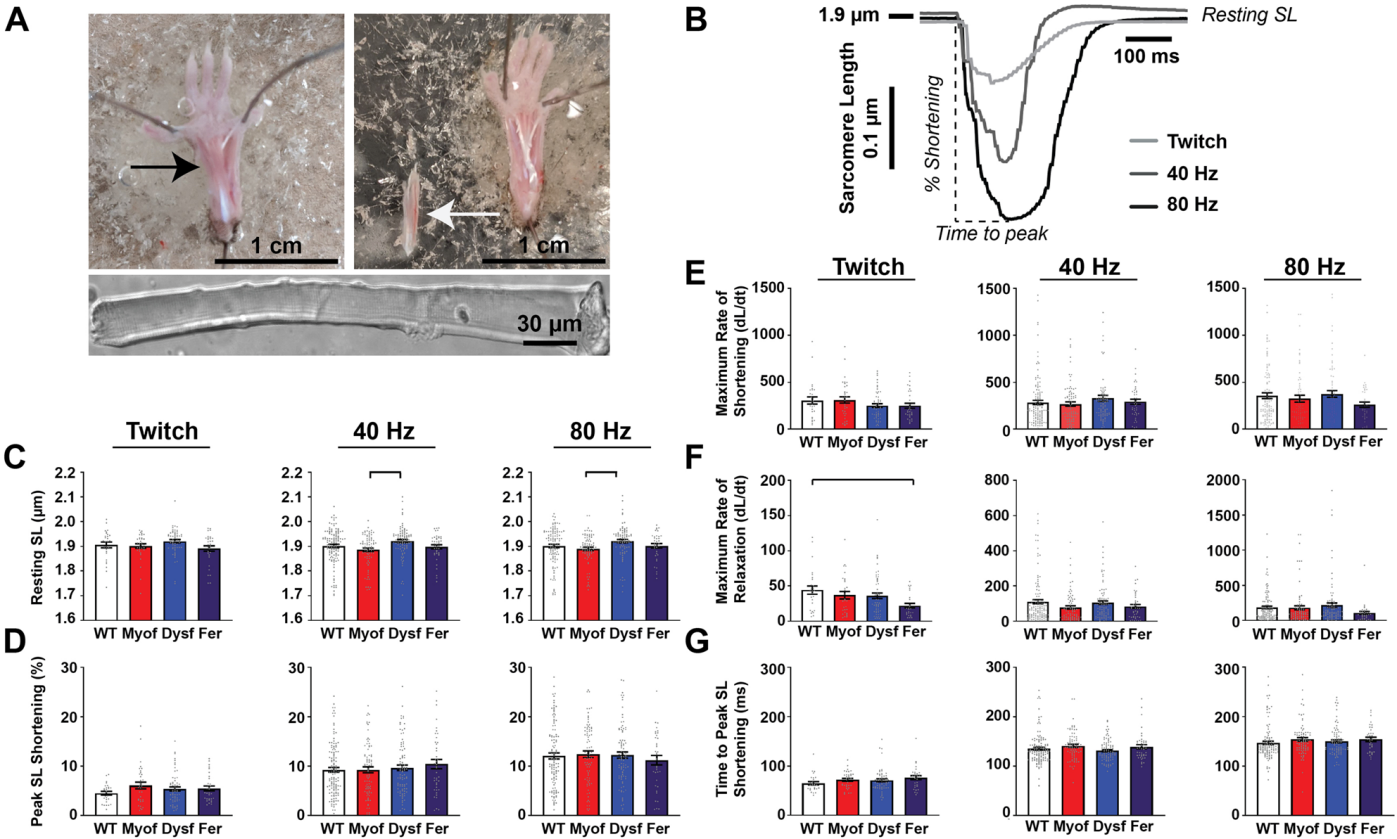
Loss of ferlin proteins cause minimal alterations in unloaded sarcomere shortening in isolated skeletal myofibers. (**A**) Depiction of mouse hind-limb footpad FDB muscle (arrows) dissection and myofiber isolation (bottom). (**B**) Representative unloaded sarcomere-length shortening curves at twitch, 40, and 80 Hz stimulation frequencies. The 80 Hz trace is marked to illustrate the parameters reported in this figure. (**C**) Resting sarcomere length, as measured during periods between pacing trains, was subtly shorter in Myof myofibers compared to Dysf at 40 and 80 Hz. (**D**) Peak % sarcomere shortening was not significantly different between groups. (**E**) The peak rate of shortening was not significantly different between groups. (**F**) Maximum rate of sarcomere relaxation was significantly slower in twitch contractions in Fer myofibers compared to WT. (**G**) The time to peak sarcomere length shortening was not significantly different between groups. For detailed N values see methods. Bar = p < 0.05 by One-Way ANOVA.

As prior work demonstrated sarcotubular disarray in Myof mice, Dysf mice or mice lacking both myoferlin and dysferlin, we assessed the functional consequences of sarcotubular disarray on excitation–contraction. Individual FDB myofibers were isolated from WT, Myof, Dysf, and Fer mice (Fig. 1A). We were interested in assessing the response of the myofibers to twitch, summating and tetanic stimuli, so we stimulated the cells at twitch, 40 Hz, and 80 Hz and measured unloaded sarcomere length shortening and calcium transients. Representative unloaded sarcomere shortening traces are shown in Fig. 1B. The resting sarcomere length, as measured in the period between stimuli trains, was significantly different between Myof and Dysf groups at 40 and 80 Hz (Fig. 1C). However, the magnitude of this change did not appear to alter peak sarcomere length shortening for any group (Fig. 1D). The maximum rate of shortening was not different between groups (Fig. 1E), whereas the maximum rate of relaxation was significantly reduced in Fer myofibers compared to WT at twitch (Fig. 1F). No significant differences in time to peak sarcomere length shortening were observed between any group (Fig. 1G). These data suggest that despite the previously identified morphological disparities in the myofibers between these genotypes, unloaded shortening was not highly affected under these conditions.

### Loss of Dysf and/or Myof is linked to differential calcium transient abnormalities in myofibers

**Figure 2:**
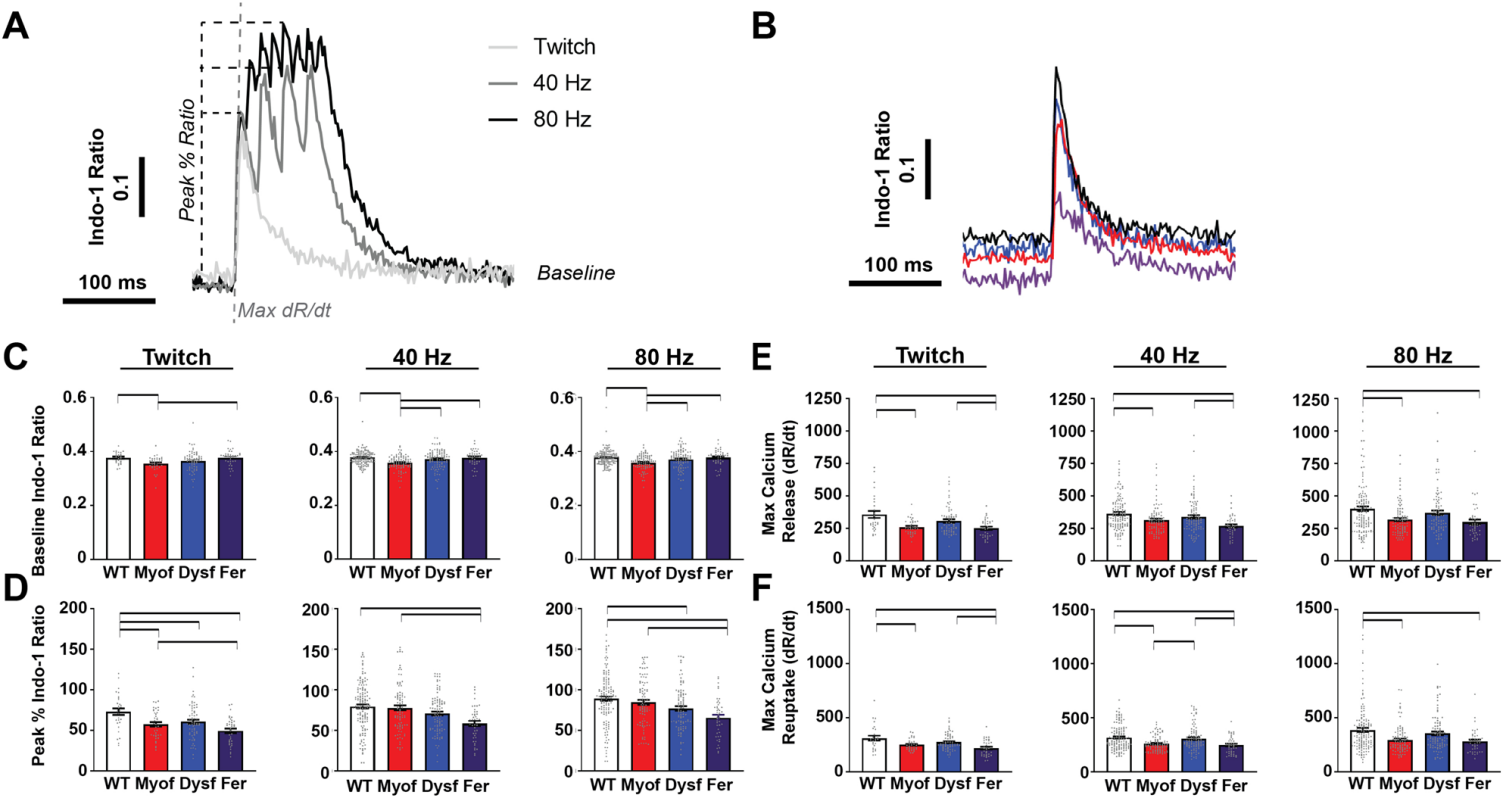
Loss of ferlin proteins is linked to differential calcium transient abnormalities in Dysf and Myof myofibers. (**A**) Representative schematic calcium transients measured by Indo-1 AM ratiometric calcium dye in isolated FDB myofibers at twitch, 40, and 80 Hz stimulation frequencies. The traces are marked to illustrate the parameters reported in this figure. (**B**) Twitch traces from WT, Myof, Dysf, and Fer myofibers. (**C**) Resting cytosolic calcium levels, as measured during periods between pacing trains, were significantly lower in Myof myofibers compared to WT and Fer groups at twitch and compared to all other groups at 40 and 80 Hz. (**D**) Peak % calcium release, which is a value of peak calcium signal normalized to resting calcium, was significantly lower in Fer myofibers compared to WT and Myof at all frequencies, and to Dysf to WT at twitch and 80 Hz. Peak % calcium release was only significantly different between Myof and WT myofibers at twitch. Dysf myofibers showed reduced peak calcium levels compared to WT at twitch and 80 Hz. (**E**) Maximum rate of calcium release measured as the change in Indo-1 ratio over time was significantly reduced in Myof and Fer myofibers compared to WT at all frequencies, were significantly different compared to Dysf only at twitch and 40 Hz. (**F**) Maximum rate of calcium reuptake was significantly reduced in Myof and Fer myofibers compared to WT at all frequencies, while Fer myofibers were significantly different compared to Dysf only at twitch and 40 Hz. For detailed N values see methods. Bar = p < 0.05 by One-Way ANOVA.

We next determined whether the disordered t-tubule network observed in mice lacking ferlin proteins was associated with defects in calcium handling. To assess this, we measured calcium transients simultaneously with our sarcomere length shortening measurements in WT, Myof, Dysf, and Fer myofibers using Indo-1^45^. Representative traces of twitch, 40, and 80 Hz stimulations are shown in Fig. 2A. Single calcium transients from twitch stimulations of WT, Myof, Dysf, and Fer myofibers are shown in Fig. 2B. Measurement of Indo-1 fluorescence revealed significantly lower resting calcium levels in Myof myofibers, measured in the periods between pacing trains, compared WT at twitch, and compared to all groups when stimulated at 40 and 80 Hz (Fig. 2C). Myofibers from Fer animals showed significantly lower peak calcium transient amplitudes compared to WT and Myof genotypes at all frequencies, whereas Dysf fibers showed reduced transient amplitude compared to WT only at twitch and 80 Hz stimulation (Fig. 2D), consistent with impaired calcium release in these muscles. The reduced peak calcium in Fer myofibers was accompanied by a slower maximum rate of calcium release compared to WT at all frequencies, and Dysf at twitch and 40 Hz. Myof fibers also showing a deficit compared to WT at all frequencies (Fig. 2E). Additionally, Fer myofibers displayed slower maximum rate of calcium reuptake compared to WT at all frequencies, and Dysf at twitch and 40 Hz. Myof fibers also showed delayed reuptake compared to WT at all frequencies (Fig. 2F). These data reveal that combined loss of both myoferlin and dysferlin results in significant impairment of calcium release.

### A greater percent of Fer and Myof myofibers showed a lower sensitivity for EC coupling

**Figure 3:**
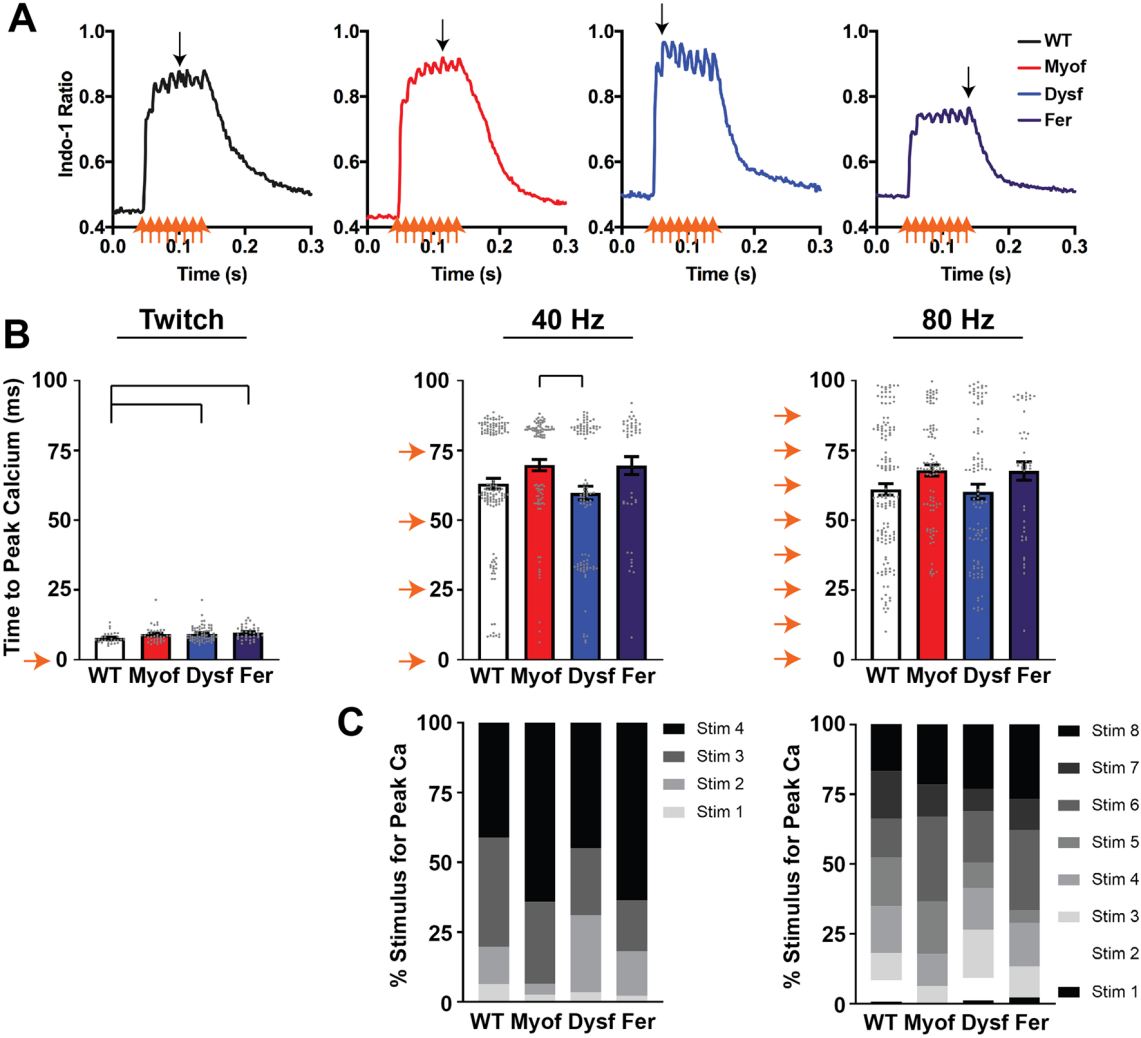
Loss of ferlin proteins resulted in differential effects on the sensitivity of calcium release to excitation. (**A**) Representative calcium transients at 80 Hz stimulation showing individual calcium release peaks, with some traces showing calcium release earlier or later during the stimulation (peak calcium marked with black arrows). (**B**) Time to peak calcium is significantly prolonged in FER myofibers compared to WT at twitch. There is a significantly reduced time to peak calcium release in Dysf myofibers compared to Myof at 40 Hz stimulation. No other significant differences are detected, although the discrete values that accompany stimulation at twitch, 40, and 80 Hz (orange arrows) can easily be seen in time-to-peak values from individual myofibers. (**C**) To assess this non-normal distribution of data, we identified the number of myofibers that reached max calcium at the 1st, 2nd, 3rd, etc. stimulation pulse at the different frequencies. WT and Dysf myofibers more often reached peak calcium at early stimuli, whereas a greater percentage of Fer and Myof myofibers reached peak calcium levels at later stimuli. For detailed N values see methods. Bar at twitch = p < 0.05 by One-Way ANOVA. Bar at 40 Hz = p < 0.05 by Kruskal–Wallis One-Way ANOVA.

We hypothesized that the triad abnormalities present in Dysf, Myof, and Fer muscle would cause aberrant coupling of excitation to calcium release^30^. To address this question, we assessed the time to peak calcium release in all four genotypes by examining the Indo-1 calcium release traces. Variation in tetanic calcium release peaks were observed across groups, with some traces showing calcium release earlier or later during the tetanic stimulations. Representative calcium transients are shown at 80 Hz stimulation, showing individual calcium release peaks and illustrating different calcium release profiles (Fig. 3A). At twitch stimulation, time to peak calcium release was significantly slower in Dysf and Fer compared to control. At tetanic stimulation frequencies, time to peak calcium was not significantly different between groups, except for Dysf myofibers that were significantly faster than Myof myofibers at 40 Hz stimulation (Fig. 3B). However, close inspection of the peak calcium times and the transients from each myofiber revealed a non-continuous, non-normal distribution of data. Each pacing stimulation and its corresponding calcium release event during tetanic stimuli can be observed in the average calcium transient (Fig. 3A). This difference in timing of the peak calcium release value can be seen in the distribution of individual time to peak data points compared with the timing of the tetanic stimuli marked on the Y-axis with orange arrows (Fig. 3B). The myofibers were stimulated once at twitch, four times at 40 Hz, and eight times at 80 Hz over a 100 ms period. Peak calcium release from an initial or early tetanic stimuli suggest a higher sensitivity to EC coupling and greater early calcium release (Fig. 3C). The higher number of stimulations required to reach peak calcium that were observed for Fer and Myo are consistent with a lower sensitivity to EC coupling. We observed that Fer and Myof myofibers had a higher percentage of peak calcium times occurring with a greater number of stimulations to reach peak calcium (Fig. 3C). These data combined suggest that loss of dysferlin generally potentiates the sensitivity of excitation to calcium release during short tetanic stimulation, whereas loss of myoferlin causes desensitization (Table 1).

**Table 1:** Summary of physiological changes compared to WT.

	Myof	Dysf	Fer
Contractility	–	–	–
Shortening kinetics	–	–	Slower
Peak calcium	Down	Down	Down
Tetanic time to peak calcium	Slower	–	Slower
Calcium kinetics	Slower	–	Slower

### Differential ryanodine receptor clustering and organization with loss of ferlin proteins

**Figure 4:**
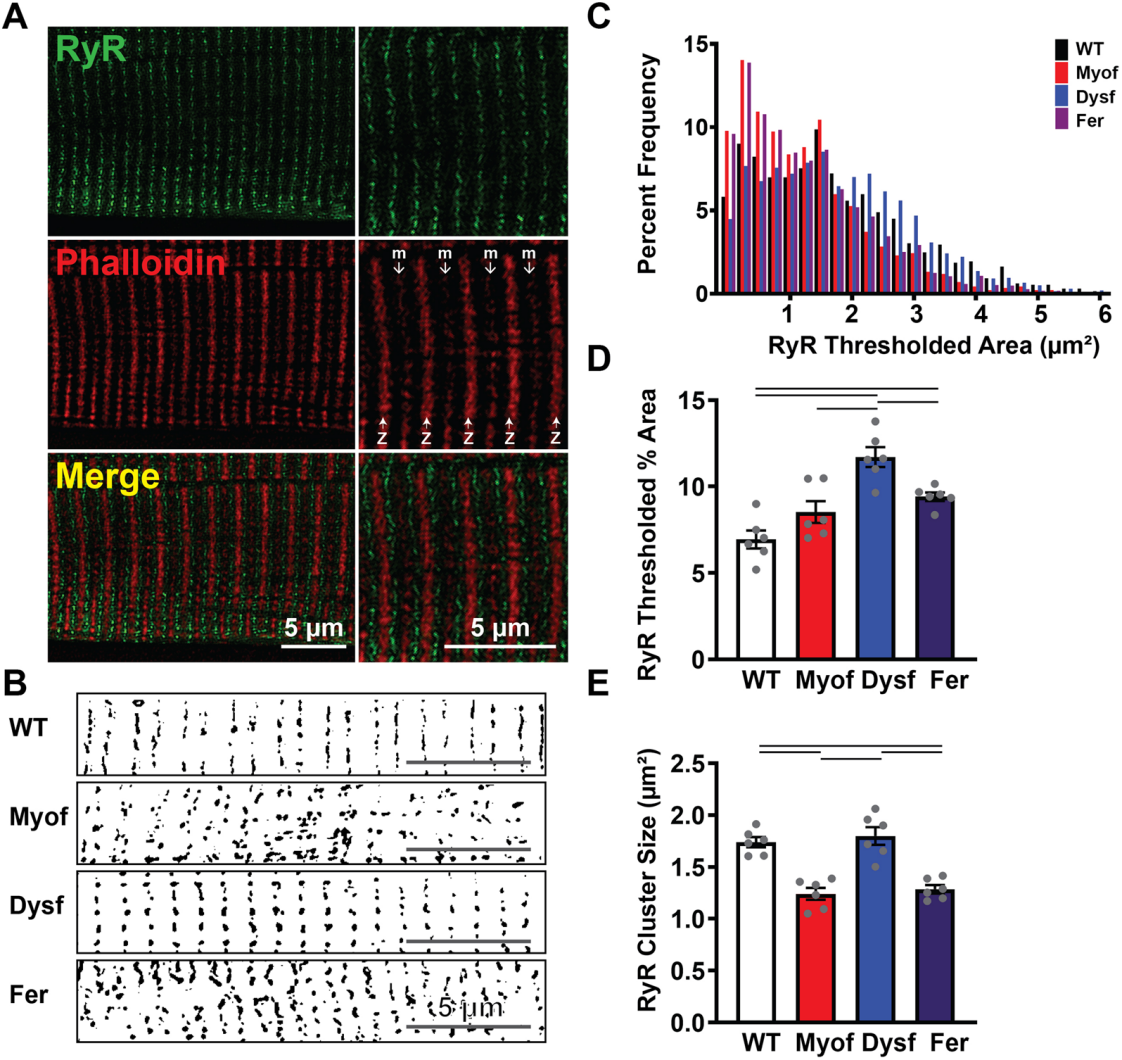
Super-resolution structured illumination microscopy (SIM) reveals abnormal ryanodine receptor cluster organization following loss of myoferlin. (**A**) Representative SIM images of anti-RyR (green) and phalloidin-stained (red) Z-disks (z, open arrow) and M-lines (m, closed arrow) in isolated FDB myofibers. (**B**) Representative Otsu thresholded SIM images show alterations in RyR clusters. (**C**) Smaller RyR cluster sizes occur at higher frequencies in Myof and Fer myofibers, with a tendency for more RyR clusters larger than 40 pixels in size in Dysf myofibers (N = 2 mice, 6 cells per group, Clusters: WT 1286; Myof 2261; Dysf 1983; Fer 2868). (**D**) RyR cluster % area is significantly higher in Dysf myofibers (N = 2 mice, 6 cells per group). (**E**) Analysis of average RyR cluster sizes following Otsu thresholding shows significantly smaller clusters in Myof and Fer fibers. (N = 2 mice, 6 cells per group). Bar = p < 0.05 by One-Way ANOVA.

Due to the functional change in calcium handling between the different genotypes, we investigated ryanodine receptor distribution, as skeletal muscle contraction is almost completely dependent on calcium outflow from the sarcoplasmic reticulum through ryanodine receptor channels^13^. Isolated FDB myofibers were immunostained with an antibody against the ryanodine receptor and imaged using a structured illuminated microscopy (SIM). Figure 4A shows the staining of ryanodine receptor clusters in WT mice, with the ryanodine receptor clusters shown in green flanking the Z-disks, marked by phalloidin, in red. SIM revealed differences in RyR organization between the genotypes (Fig. 4B). Image thresholding and quantification of total RyR fluorescence area showed a general increase in the total area occupied by RyR clusters in Dysf myofibers compared to all groups (Fig. 4C–E). Total RyR fluorescence area was significantly increased in FER myofibers compared to WT (Fig. 4D). Quantification of the individual cluster size showed significantly smaller average cluster size in Myof and Fer fibers (Fig. 4E). While the group average was not different between Dysf and WT, examination of the size distribution shows that myofibers from Dysf mice had a higher abundance of thresholded regions 2 µm^2^ or larger compared to WT (Fig. 4C). Overall, these data suggest that ferlin proteins play differential roles in organization of ryanodine receptor clusters which may, in part, explain differences in calcium handling within myofibers.

**Figure 5:**
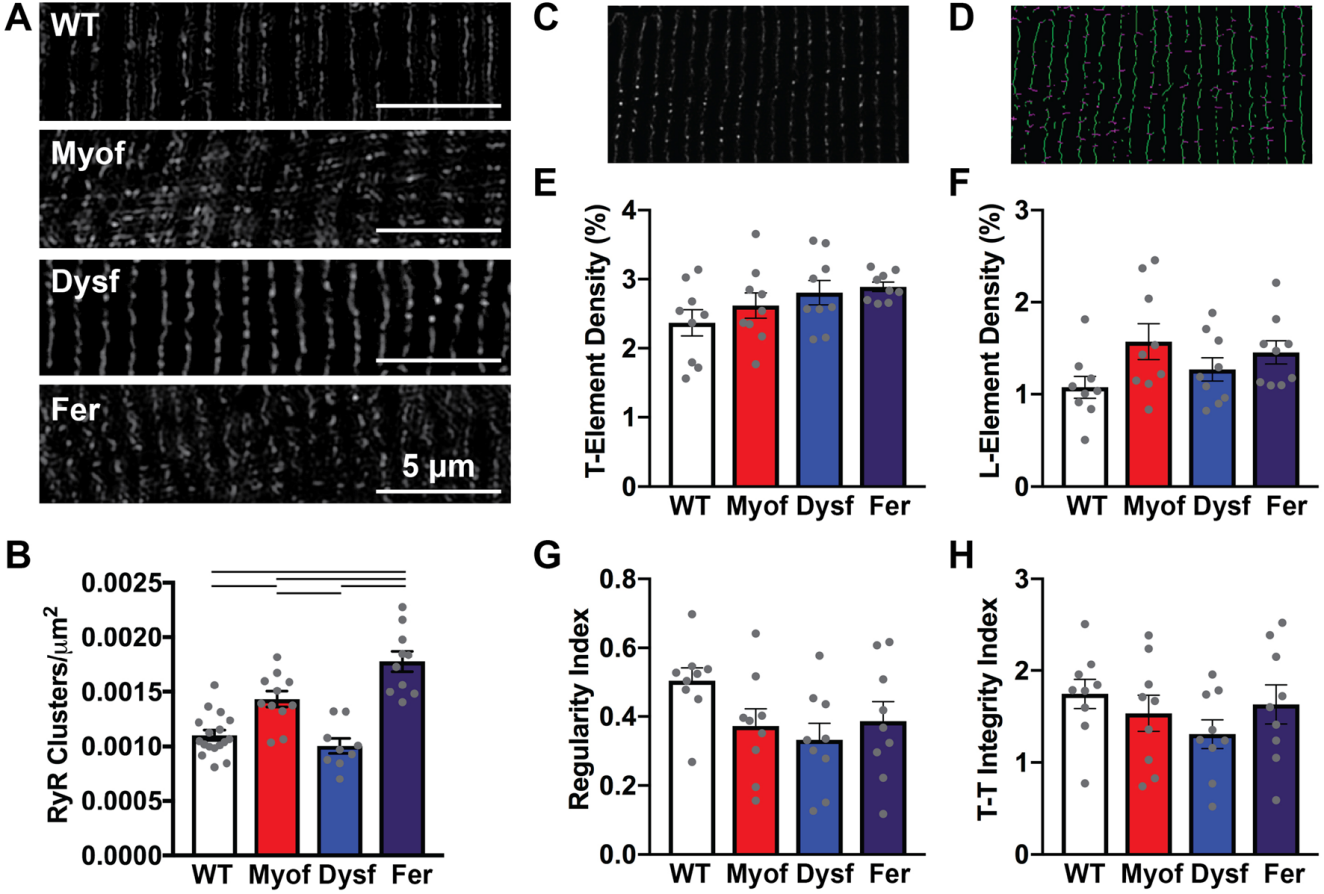
Loss of myoferlin resulted in reduced RyR cluster regularity and improper localization. (**A**) Representative SIM images of anti-RyR FDB myofiber staining in all genotypes. (**B**) RyR cluster counts per area are significantly higher in Myo and Fer myofibers than WT and Dysf (N = WT 3 mice, 17 fibers; Myof 3 mice, 11 fibers; Dysf 3 mice, 9 fibers; Fer 3 mice, 10 fibers). (**C**, **D**) Representative SIM image of RyR staining in isolated FDB myofibers and a corresponding thresholded, skeletonized image used for Auto-TT analysis of RyR cluster organization. (**E**) Transverse (t) RyR clusters were not significantly changed between genotypes. (**F**) There were non-significant elevations and high variability in the amount of aberrant longitudinally-oriented RyR clusters in Myof, Dysf, and Fer myofibers. (**G**) An overall cellular regularity index of RyR cluster, and the integrity of T-T spacing shows non-significant reduction in Myof and Fer compared to WT and Dysf myofibers. N = 3 mice, 3 fibers per mouse.

We then evaluated the subcellular organization of RyR clusters. SIM images were used to count the number of RyR clusters per µm^2^ by a blinded observed and showed significantly higher RyR cluster density in Fer and Myof myofibers compared to WT and Dysf (Fig. 5A,B). To quantify ryanodine receptor cluster organizational parameters, we utilized the AutoTT program that was developed for the analysis of T-tubule regularity^46^. AutoTT outputs the densities of the transversely (T) and longitudinally (L) oriented elements, providing information on the orientation of groups of RyR clusters (Fig. 5C,D). No significant differences were found in transverse T- or L-element density between genotypes (Fig. 5E,F). AutoTT analysis provided quantification of the global regularity of the RyR cluster organization based on a Fourier transform, and did not show significant differences between groups (Fig. 5G). A measure of the integrity of the T-element spacing also showed high variability among groups and no significant differences between groups (Fig. 5H).

## Discussion

### Ferlin proteins are required for normal t-tubule and triad formation in skeletal myofibers

We previously demonstrated that mouse muscle lacking dysferlin or myoferlin exhibit dystrophic muscle morphology and sarcotubular disarray, with the loss of both myoferlin and dysferlin resulting in a more severe muscular dystrophy phenotype^30^. Specifically, the combined loss of myoferlin and dysferlin was characterized by highly disorganized triad structures and sarcotubular aggregates. As defects in triad formation leading to muscle weakness and excitation–contraction coupling have been previously reported in models lacking mitsugumin-29 or junctophilin 1^19,47,48^, we hypothesized that the histological defects observed in mice lacking ferlin proteins could lead to altered calcium regulation and aberrant EC coupling. Herein, we show that the loss of myoferlin or dysferlin results in distinctly different pathophysiology.

### Triad formation and dysfunction

Transverse tubule development is incompletely understood, but a number of essential components have been identified. Caveolin 3 (*Cav3*) is necessary for proper t-tubule formation and is thought to create initial caveolae that extend into mature t-tubules^49–52^. Loss of function mutations in *CAV3* have been described in limb-girdle muscular dystrophy type 1C and rippling muscle disease, an unusual disorder of hypercontraction^49,53–55^. Dysferlin binds to Cav3 during development of t-tubules^56,57^.

Several proteins interact and regulate the triad space, interacting with t-tubule and junctional SR proteins, including dysferlin^19,47,58–62^. Junctophilin-1 and -2 hetero or homodimers to the junctional SR via a single carboxy-terminal transmembrane domain and also bind to the t-tubule via their amino-terminal cytosolic domain^61,62^. Mice lacking functional junctophilin-1 exhibit normally formed muscle, but with fewer triads that are mislocalized to the A/I junction, and functionally have a decreased sensitivity of the force-frequency relationship and die perinatally^47^. This is similar to the SR malformation in the Myof and Fer mice that we previously reported^30^. In addition to physically linking these two triad components, junctophilin can interact with the carboxy-terminus of the DHPR (Cav1.1), and mice with mutations in these interacting sites exhibit impaired DHPR coupling with the RyR and impaired muscle force development^63^. Impaired EC coupling and t-tubule dissary following the loss of junctophilin mirrors the phenotype we observe in these experiments and in the prior morpholigcal analysis^19,30,47^.

### Skeletal muscle calcium handling and RyR organization

Mammalian skeletal muscle excitation–contraction coupling is mediated through conformational coupling that link the DHPR α_1S_ subunit to the ryanodine receptor ion channel^5,11,14^. Previous studies have also provided data on potential interactions between dysferlin and the DHPR receptor^29,59^; however, the direct effect of ferlin proteins regulating RyR clusters have not been reported. We studied ryanodine receptor-1 in FDB myofibers from mice lacking ferlin proteins to help explain the differences identified in calcium handling and sensitivity of EC coupling. We showed that the RyR cluster count per µm^2^ was higher in Myof and Fer myofibers while the clusters themselves were smaller in size and less organized compared to Dysf and WT clusters. The morphological changes in these fibers and the known interaction of dysferlin with DHPR points to the possibility that t-tubule, triad, and SR developmental disorganization is fragmenting functional DHPR-RyR cluster complexes and reducing the calcium release response to stimuli in Myof and Fer myofibers. These data, complementary to the data published by Demonbreun et al. in 2014, indicates differential roles for ferlin proteins in sarcotubular formation and unique impairments based on which ferlin protein is absent. Although *MYOF* mutations have not been described in human muscular dystrophy, we show functional consequences for mature mouse muscle lacking myoferlin. It is feasible that mutations in myoferlin may act in conjunction with primary disease-causing mutations, such as those found in dysferlin, to modify and exacerbate the disease phenotype. Further studies are required to assess the contribution of myoferlin mutations in the context of human muscle disease.

The loss of dysferlin alone showed normal to increased sensitivity to EC coupling during tetanic stimulation, regularly organized ryanodine receptors, and a ryanodine receptor % area that is larger than those observed in WT myofibers. While the average RyR cluster size was not different between Dysf and WT, Dysf myofibers had a greater number of RyR thresholded regions 2 µm^2^ or larger compared to WT. Alterations in ryanodine receptor-2 cluster size has been shown to have functional impacts on spark frequency in cardiac myocytes^16^. In skeletal muscle, however, the functional consequences of altered ryanodine receptor clustering remain unclear. Other work has shown that dysferlin and DHPR co-localize and that dysferlin may be involved in the formation of an oligomeric complex with DHPR and caveolin-3^29,59^. In 2017, Lukyanenko et al. published data suggesting that dysferlin modulates SR calcium release in skeletal muscle and that in absence of dysferlin, osmotic shock injury results in increased ryanodine receptor-1-mediated calcium leak from the SR into the cytoplasm^5^. We propose a hypothetical model where defects in membrane fusion and development in Myof and Fer myofibers, due primarily to the loss of myoferlin, leads to defective triad formation and dysfunctional EC coupling. As myoferlin is still expressed in Dysf myofibers, we hypothesize that loss of dysferlin only leads to triad disorganization and alterations in DHPR-RyR cluster organization, with overall preserved function.

### Considerations and limitations

For these experiments we used an isolated myofiber approach that provided an efficient way to measure contractile and calcium cycling properties but does omit several physiological contexts that normally influence muscle function. Of note, isolated myofibers have a resting sarcomere length around 1.9 µm, which is lower than the in situ resting length of 2.1 – 2.2 µm and will result in these fibers operating on the lower slope of the force–length relationship^64,65^. Additionally, these isolated myofibers are activated by field stimulation and not direct innervation which has been shown to result in structural remodeling of the t-tubular system^66^. The functional effect of field stimulation is likely an overactivation of the fibers and may obscure some of the activation deficits caused by disorganized T-tubule systems that would be present in vivo. However, the defined time post-isolation should control for any changes due to denervation and fiber isolation between groups in this study.

Previous work has evaluated calcium cycling dysfunction in the dysferlin null mouse originally identified on the A/J inbred mouse strain^4,5^. These data showed a ~ 20% reduction in calcium transient amplitude in isolated FDB myofibers from dysferlin null A/J mice using Rhod-2 calcium responsive dye and line scanning confocal measurements^5^, whereas Kerr et al. 2013 used Indo-1 in isolated A/J FDB myofibers and showed no difference in peak calcium release following tetanic stimulation. The reason for this discrepancy is unclear, but the data in the present work using Indo-1 is in agreement with the comparable method and resulting data reported in Kerr et al. 2013. Of note, these prior studies used the A/J inbred mouse lacking *Dysf*^4,5^. In this current work and our previous data^30^, we have backcrossed the *Dysf* null allele onto the 129 background to allow comparisons between Dysf, Myof, and Fer genotypes on an isogenic background, and this change in background may account for some of the functional differences observed.

There have been many studies that have used super resolution microscopy and image processing, including point spread function analysis, to report quantized, nanoscale ryanodine receptor number^16^, area, and/or discrete receptor numbers per cluster^67^. For our approach, we were not able to process the image data in this way, and instead used image thresholding to identify ryanodine receptor clusters and report the area of this thresholded signal. This approach has reduced resolution, so we have not reported RyR cluster area, but rather thresholded image area.

## Materials and methods

### Animal models

Fer mice have been previously generated by backcrossing dysferlin-null (Dysf) mice from the A/J strain for more than six generations to the 129/SVa emst/J myoferlin-null mouse line^30,40,44^. The myoferlin-null (Myof) allele was previously generated on the 129/SvEms/J as described in Doherty et al. 2005. To allow the pathophysiology of ferlin protein loss to fully manifest, the mice used in this study were between 4 and 6 months of age. Mice were euthanized by sedation with vaporized isoflurane flowed by cervical dislocation. All mice were housed, monitored, and cared for humanely in a AAALAC accredited facility in accordance with the Guide for the Care and Use of Laboratory Animals, in compliance with ARRIVE guidelines, and all procedures were approved by Northwestern University’s Institutional Animal Care and Use Committee regulations.

### FDB preparation and calcium indicator dye loading

Following euthanasia, FDB muscles were dissected from mouse hind-limb and placed in DMEM (HyClone SH30022.01) with 0.2% bovine serum albumin (Sigma-Aldrich SLBT0167) and 4 mg/mL collagenase II (Invitrogen 17101-015) solution in a 37 °C incubator with 10% CO_2_ for 90 minutes^68^. Gross imaging of the FDB muscle was obtained using a Google Pixel 3a camera. Digested FDB muscle was transferred to Ringer’s solution (123 mM NaCl, 2 mM CaCl_2_, 5 mM KCl, pH 7.4) where it was gently triturated with a 1000 µL pipette tip with the tip cut off until the bulk of the tissue had been disrupted, leaving single cells. Glass bottom microwell dishes (MatTek) were coated with 20 µg/mL laminin (Gibco 23017-015) and allowed to incubate at room temperature for one hour prior to use. The isolated FDB myofibers were adhered to the laminin coated glass bottom microwell dishes for one hour at 37 °C maintained in an incubator with 5% CO_2_. The media was then changed to 300 µL of serum-free DMEM (Gibco 11995073) with 1% penicillin/streptomycin and cultured at 37 °C with 5% CO_2_ to allow for overnight recovery of the myofibers. Cells were isolated in the evening and experiments were performed in the morning the following day. This time frame was chosen because freshly isolated FDBs showed limited responsiveness to stimulation directly after isolation, whereas overnight recovery resulted in nearly every surviving cell having a robust physiological response. Gross imaging of isolated myofibers was performed on an EVOS Floid imaging system (ThermoFisher). FDB muscles were loaded with 5 µM Indo-1-AM (TEF Labs 145) cytosolic calcium indicator dye resuspended in DMSO and 10% Pluronic for 45 min in a 37 °C incubator with 5% CO_2_. Inside the cells, Indo-1-AM is desterified to the ratiometric calcium indicator Indo-1 that is excited at 360 nm and emits at 405 nm when bound to calcium and 475 nm when not bound to calcium^45,69^. Data was reported as the ratio of bound/unbound calcium.

### Calcium transient and sarcomere shortening measurements

To measure calcium transients and sarcomere shortening, isolated FDB muscles were imaged on a Nikon Diaphot inverted microscope equipped with a high-speed camera and photomultiplier tubes integrated with the FluoroDaq system (IonOptix, Westwood, MA) as previously described^70^. FDB muscles were paced in a custom insert with a pair of parallel platinum electrodes 9 mm apart designed for 35 mm MatTek dishes and connected to a high-voltage follow stimulator (701C Aurora Scientific, Aurora, ON, Canada). Cells were paced at twitch, 40, and 80 Hz for 100 ms using a 0.2 ms pulse width at 18–20 V (Kerr et al. 2015). Each pacing train was repeated 10 times over 20 s and were averaged for a single value per frequency per fiber and measurements at all frequencies were performed in under 5 min per cell. All measurements were made at room temperature, which was monitored and remained between 20 and 22 °C. To visualize unloaded sarcomere shortening, a video sarcomere length system (900B, Aurora Scientific) was used to measure sarcomere spacing from bright-field images using a fast-Fourier transform. FDBs were allowed to freely shorten without external load as previously described^64^. Aurora Scientific’s 950A calcium fluorescence program was used to record the calcium transients and sarcomere length shortening data and analyze the parameters of average transients. We plated FDB fibers from each mouse onto three separate plates and planned to collect data from a maximum of five fibers per plate. Any isolations where we were unable to measure more than 2 fibers per plate were considered failed isolations and the data were not used. Data were reported as N = 1 fiber as previously reported^5,64,71^, with equitable numbers of fibers from each individual animal to not bias the group towards any one isolation. The number of fibers reported in Figs. 1, 2 and 3 are Twitch N = WT: 3 mice, 6, 14, 9 fibers each, 29 total fibers; Myof: 3 mice, 14, 14, 9 fibers each, 37 total fibers; Dysf: 6 mice, 10, 9, 9, 12, 14, 14 fibers each, 68 total fibers; Fer: 3 mice, 12, 13, 12 fibers each, 37 total fibers. For 40 & 80 Hz, N = WT: 11 mice, 14, 12, 14, 15, 15 , 9, 10, 10, 15, 13, 15 fibers each, 142 total fibers; Myof: 7 mice, 15, 14, 14, 7, 15, 15, 15 fibers each, 95 total fibers; Dysf: 7 mice, 15, 15, 15, 14, 15, 15, 15 fibers each, 104 total fibers; Fer: 3 mice, 15, 15, 15 fibers each, 45 total fibers.

### Immunostaining and immunofluorescence microscopy

Following plating of isolated FDB myofibers on laminin-coated 10 mm diameter glass coverslips for 1 h at 37 °C, FDB myofibers were washed once with 1 mL of phosphate-buffered saline solution. The cells were then incubated in 4% paraformaldehyde diluted in PBS for 15 min at 4 °C. Fibers were permeabilized by replacing the fixation solution with PBS containing 0.25% Triton X-100 for 20 min at 4 °C. The cells were blocked with PBS containing 20% fetal bovine serum and 0.1% Triton X-100 for 30 min at 4 °C. Primary antibodies were diluted in PBS containing 2% FBS and 0.1% Triton X-100 and incubated with the cells at 4 °C overnight. Anti-ryanodine receptor antibody (Abcam GR3250452-2) was used as the primary antibody at a 1:1000 dilution. Alexa Fluor 488 goat anti-mouse IgG1 (Molecular Probes #2,040,297) was used as the secondary antibody and Alexa Fluor 568-conjugated phalloidin was used to stain actin filaments, both at a 1:1000 dilution. FDB myofibers were incubated with secondary antibody and phalloidin for one hour at 4 °C. Samples were washed three times for 10 min each in cold PBS with 0.1% Triton X-100. The second wash contained 1:1000 diluted Hoechst 3342 dye (Thermo H3570) to stain nuclei. The coverslips were mounted on glass slides with ProLong Gold (Invitrogen Cat. #1,964,378).

Super-resolution microscopy was performed using a Nikon Structured Illumination Microscopy (N-SIM) imaging platform with 488 nm and 561 nm wavelength lasers in the Nikon Imaging Center in Northwestern University’s Center for Advanced Microscopy. The N-SIM was fitted with a 100 × oil objective (NA 1.49). This configuration allowed a lateral resolution of 115 nm and an axial resolution of 296 nm, with 65 nm^2^ pixel size. Images were taken 5 – 10 µm deep in the fiber where transverse elements were uniformly distributed across the width of the cell, this excludes sarcolemmal and immediately subsarcolemmal RyR clusters. All images were acquired and reconstructed using the Nikon Elements software v4.50.

### Ryanodine receptor count/µm^2^ and cluster size quantification

The acquired super-resolution images were analyzed using the open source image processing program ImageJ FIJI (NIH, November 2019 release, https://imagej.net). Ryanodine receptor clusters per µm^2^ from representative sections of each FDB imaged were counted by hand. Each dot in the immunostained image represents a cluster of ryanodine receptors. At least ten FDB myofibers per genotype taken from at least three preparations were analyzed. Percent area was calculated on images subjected to Otsu thresholding in ImageJ. The percentage of ryanodine receptor fluorescence signal in the total area were measured and the pixels in each ryanodine receptor cluster were counted to compare the average ryanodine receptor cluster size between the different genotypes.

### Ryanodine receptor distribution quantification

To measure the ryanodine receptor cluster organization, the AutoTT program in MatLab v2015a was used. AutoTT is an automated analysis program that combines image processing, morphological feature extraction, and fast Fourier transformation analysis of spectrum power^46^. AutoTT converts an image to a binary image then subsequently skeletonizes it. The resulting binary and skeletonized images were subjected to morphological feature analysis to provide the densities of the transversely and longitudinally oriented elements and the global regularity of the tubules.

### Statistical analysis

Statistical evaluation was performed using GraphPad Prism7. P < 0.05 was considered statistically significant. Data is presented as mean ± standard error or the mean with individual data points overlaid. All data was assessed for normalcy with a Bartlett’s test and groups were compared using a One-Way ANOVA with a Holm-Sidak multiple comparison post-hoc test. Non-parametric data in Fig. 3 was analyzed with a Kruskal–Wallis one-way ANOVA.

## Abbreviations

WT: Wild-type
Myof: Myoferlin-null mouse
Dysf: Dysferlin-null mouse
Fer: Ferlin-null mouse
EC: Excitation–contraction
SR: Sarcoplasmic reticulum
DHPR: Dihydropyridine receptor
RyR: Ryanodine receptor
FDB: Flexor digitorum brevis
SL: Sarcomere length

## References

[1] Dalkilic I, Kunkel LM (2003). Muscular dystrophies: genes to pathogenesis. Curr. Opin. Genet. Dev..

[2] Duan D, Goemans N, Takeda S, Mercuri E, Aartsma-Rus A (2021). Duchenne muscular dystrophy. Nat. Rev. Dis. Primers.

[3] Emery AE (1998). The muscular dystrophies. BMJ.

[4] Kerr JP (2013). Dysferlin stabilizes stress-induced Ca2+ signaling in the transverse tubule membrane. Proc. Natl. Acad. Sci. USA.

[5] Lukyanenko V, Muriel JM, Bloch RJ (2017). Coupling of excitation to Ca(2+) release is modulated by dysferlin. J. Physiol..

[6] Burr AR, Molkentin JD (2015). Genetic evidence in the mouse solidifies the calcium hypothesis of myofiber death in muscular dystrophy. Cell Death Differ..

[7] Jungbluth H, Gautel M (2014). Pathogenic mechanisms in centronuclear myopathies. Front. Aging Neurosci..

[8] Capote J, DiFranco M, Vergara JL (2010). Excitation-contraction coupling alterations in mdx and utrophin/dystrophin double knockout mice: a comparative study. Am. J. Physiol. Cell Physiol..

[9] Mareedu S, Million ED, Duan D, Babu GJ (2021). Abnormal calcium handling in duchenne muscular dystrophy: mechanisms and potential therapies. Front. Physiol..

[10] Vallejo-Illarramendi A, Toral-Ojeda I, Aldanondo G, Lopez de Munain A (2014). Dysregulation of calcium homeostasis in muscular dystrophies. Expert Rev. Mol. Med..

[11] Rebbeck RT (2014). Skeletal muscle excitation-contraction coupling: who are the dancing partners?. Int. J. Biochem. Cell Biol..

[12] Calderon JC, Bolanos P, Caputo C (2014). The excitation-contraction coupling mechanism in skeletal muscle. Biophys. Rev..

[13] Nakai J (1997). Functional nonequality of the cardiac and skeletal ryanodine receptors. Proc. Natl. Acad. Sci. USA.

[14] Polster A, Nelson BR, Papadopoulos S, Olson EN, Beam KG (2018). Stac proteins associate with the critical domain for excitation-contraction coupling in the II–III loop of CaV11. J. Gen. Physiol..

[15] Block BA, Imagawa T, Campbell KP, Franzini-Armstrong C (1988). Structural evidence for direct interaction between the molecular components of the transverse tubule/sarcoplasmic reticulum junction in skeletal muscle. J. Cell Biol..

[16] Galice S, Xie Y, Yang Y, Sato D, Bers DM (2018). Size matters: ryanodine receptor cluster size affects arrhythmogenic sarcoplasmic reticulum calcium release. J. Am. Heart Assoc..

[17] Kerr JP, Ward CW, Bloch RJ (2014). Dysferlin at transverse tubules regulates Ca(2+) homeostasis in skeletal muscle. Front. Physiol..

[18] Marty I, Faure J (2016). Excitation-contraction coupling alterations in myopathies. J. Neuromuscul. Dis..

[19] Al-Qusairi L, Laporte J (2011). T-tubule biogenesis and triad formation in skeletal muscle and implication in human diseases. Skelet. Muscle.

[20] Dirksen RT, Avila G (2002). Altered ryanodine receptor function in central core disease: leaky or uncoupled Ca(2+) release channels?. Trends Cardiovasc. Med..

[21] Demonbreun AR (2011). Impaired muscle growth and response to insulin-like growth factor 1 in dysferlin-mediated muscular dystrophy. Hum. Mol. Genet..

[22] Bashir R (1998). A gene related to *Caenorhabditis elegans* spermatogenesis factor fer-1 is mutated in limb-girdle muscular dystrophy type 2B. Nat. Genet..

[23] Davis DB, Doherty KR, Delmonte AJ, McNally EM (2002). Calcium-sensitive phospholipid binding properties of normal and mutant ferlin C2 domains. J. Biol. Chem..

[24] Lek A, Evesson FJ, Sutton RB, North KN, Cooper ST (2012). Ferlins: regulators of vesicle fusion for auditory neurotransmission, receptor trafficking and membrane repair. Traffic.

[25] Posey AD, Demonbreun A, McNally EM (2011). Ferlin proteins in myoblast fusion and muscle growth. Curr. Top. Dev. Biol..

[26] Anderson LV (1999). Dysferlin is a plasma membrane protein and is expressed early in human development. Hum. Mol. Genet..

[27] Gayathri N (2011). Dysferlinopathy: spectrum of pathological changes in skeletal muscle tissue. Indian J. Pathol. Microbiol..

[28] Liu J (1998). Dysferlin, a novel skeletal muscle gene, is mutated in Miyoshi myopathy and limb girdle muscular dystrophy. Nat. Genet..

[29] Ampong BN, Imamura M, Matsumiya T, Yoshida M, Takeda S (2005). Intracellular localization of dysferlin and its association with the dihydropyridine receptor. Acta Myol..

[30] Demonbreun AR (2014). Dysferlin and myoferlin regulate transverse tubule formation and glycerol sensitivity. Am. J. Pathol..

[31] Bittner RE (1999). Dysferlin deletion in SJL mice (SJL-Dysf) defines a natural model for limb girdle muscular dystrophy 2B. Nat. Genet..

[32] Kobayashi K, Izawa T, Kuwamura M, Yamate J (2012). Dysferlin and animal models for dysferlinopathy. J. Toxicol. Pathol..

[33] Bansal D (2003). Defective membrane repair in dysferlin-deficient muscular dystrophy. Nature.

[34] McDade JR, Michele DE (2014). Membrane damage-induced vesicle-vesicle fusion of dysferlin-containing vesicles in muscle cells requires microtubules and kinesin. Hum. Mol. Genet..

[35] Klinge L (2010). Dysferlin associates with the developing T-tubule system in rodent and human skeletal muscle. Muscle Nerve.

[36] Klinge L (2007). From T-tubule to sarcolemma: damage-induced dysferlin translocation in early myogenesis. Faseb J..

[37] Redpath GMI, Sophocleous RA, Turnbull L, Whitchurch CB, Cooper ST (2016). Ferlins show tissue-specific expression and segregate as plasma membrane/late endosomal or trans-golgi/recycling ferlins. Traffic.

[38] Waddell LB (2011). Dysferlin, annexin A1, and mitsugumin 53 are upregulated in muscular dystrophy and localize to longitudinal tubules of the T-system with stretch. J. Neuropathol. Exp. Neurol..

[39] Davis DB, Delmonte AJ, Ly CT, McNally EM (2000). Myoferlin, a candidate gene and potential modifier of muscular dystrophy. Hum. Mol. Genet..

[40] Doherty KR (2005). Normal myoblast fusion requires myoferlin. Development.

[41] Han S (2019). Myoferlin regulates Wnt/β-catenin signaling-mediated skeletal muscle development by stabilizing dishevelled-2 against autophagy. Int. J. Mol. Sci..

[42] Doherty KR (2008). The endocytic recycling protein EHD2 interacts with myoferlin to regulate myoblast fusion. J. Biol. Chem..

[43] Demonbreun AR (2010). Myoferlin regulation by NFAT in muscle injury, regeneration and repair. J. Cell Sci..

[44] Ho M (2004). Disruption of muscle membrane and phenotype divergence in two novel mouse models of dysferlin deficiency. Hum. Mol. Genet..

[45] Bannwarth M (2009). Indo-1 derivatives for local calcium sensing. ACS Chem. Biol..

[46] Guo A, Song LS (2014). AutoTT: automated detection and analysis of T-tubule architecture in cardiomyocytes. Biophys. J..

[47] Ito K (2001). Deficiency of triad junction and contraction in mutant skeletal muscle lacking junctophilin type 1. J. Cell Biol..

[48] Komazaki S, Nishi M, Takeshima H, Nakamura H (2001). Abnormal formation of sarcoplasmic reticulum networks and triads during early development of skeletal muscle cells in mitsugumin29-deficient mice. Dev. Growth Differ..

[49] Gazzerro E, Sotgia F, Bruno C, Lisanti MP, Minetti C (2010). Caveolinopathies: from the biology of caveolin-3 to human diseases. Eur. J. Hum. Genet..

[50] Hernández-Deviez DJ (2006). Aberrant dysferlin trafficking in cells lacking caveolin or expressing dystrophy mutants of caveolin-3. Hum. Mol. Genet..

[51] Parton RG, Way M, Zorzi N, Stang E (1997). Caveolin-3 associates with developing T-tubules during muscle differentiation. J. Cell Biol..

[52] Galbiati F (2001). Caveolin-3 null mice show a loss of caveolae, changes in the microdomain distribution of the dystrophin-glycoprotein complex, and T-tubule abnormalities *. J. Biol. Chem..

[53] Minetti C (1998). Mutations in the caveolin-3 gene cause autosomal dominant limb-girdle muscular dystrophy. Nat. Genet..

[54] McNally EM (1998). Caveolin-3 in muscular dystrophy. Hum. Mol. Genet..

[55] Kubisch C (2003). Homozygous mutations in caveolin-3 cause a severe form of rippling muscle disease. Ann. Neurol..

[56] Capanni C (2003). Dysferlin in a hyperCKaemic patient with caveolin 3 mutation and in C2C12 cells after p38 MAP kinase inhibition. Exp. Mol. Med..

[57] Matsuda C (2001). The sarcolemmal proteins dysferlin and caveolin-3 interact in skeletal muscle. Hum. Mol. Genet..

[58] Takeshima H (1998). Mitsugumin29, a novel synaptophysin family member from the triad junction in skeletal muscle. Biochem. J..

[59] Kerr JP (2013). Dysferlin stabilizes stress-induced Ca2+ signaling in the transverse tubule membrane. Proc. Natl. Acad. Sci..

[60] Hofhuis J (2017). Dysferlin mediates membrane tubulation and links T-tubule biogenesis to muscular dystrophy. J. Cell Sci..

[61] Takeshima H, Komazaki S, Nishi M, Iino M, Kangawa K (2000). Junctophilins: a novel family of junctional membrane complex proteins. Mol. Cell.

[62] Rossi D (2019). Molecular determinants of homo- and heteromeric interactions of Junctophilin-1 at triads in adult skeletal muscle fibers. Proc. Natl. Acad. Sci. USA.

[63] Nakada T (2018). Physical interaction of junctophilin and the CaV11 C terminus is crucial for skeletal muscle contraction. Proc. Natl. Acad. Sci. USA.

[64] Kerr JP (2015). Detyrosinated microtubules modulate mechanotransduction in heart and skeletal muscle. Nat. Commun..

[65] Huxley AF, Simmons RM (1971). Proposed mechanism of force generation in striated muscle. Nature.

[66] Sellin LC, Thesleff S (1980). Alterations in membrane electrical properties during long-term denervation of rat skeletal muscles. Acta Physiol. Scand..

[67] Hou Y, Jayasinghe I, Crossman DJ, Baddeley D, Soeller C (2015). Nanoscale analysis of ryanodine receptor clusters in dyadic couplings of rat cardiac myocytes. J. Mol. Cell Cardiol..

[68] Demonbreun AR, McNally EM (2015). DNA electroporation, isolation and imaging of myofibers. J. Vis. Exp..

[69] Cheng AJ, Westerblad H (2017). Mechanical isolation, and measurement of force and myoplasmic free [Ca(2+)] in fully intact single skeletal muscle fibers. Nat. Protoc..

[70] Kim EY (2019). Distinct pathological signatures in human cellular models of myotonic dystrophy subtypes. JCI Insight.

[71] Vetter AD (2018). TnI structural interface with the N-terminal lobe of TnC as a determinant of cardiac contractility. Biophys. J..

